# Change in Mean Height of Thai Military Recruits From 1972 Through 2006

**DOI:** 10.2188/jea.JE20081018

**Published:** 2009-07-05

**Authors:** Sam-ang Seubsman, Adrian C. Sleigh

**Affiliations:** 1 School of Human Ecology and Thai Health Promotion Centre, Sukhothai Thammathirat Open University, Nonthaburi, Thailand; 2 National Centre for Epidemiology and Population Health, The Australian National University, Canberra, Australia

**Keywords:** adult height, Thailand, military recruits, health transition

## Abstract

**Background:**

Records in Western countries reveal that adult height has been increasing over the last 250 years. These height gains have been biologically associated with healthier childhoods, less illness, and longer life spans—a health-risk transition. To measure such progress in Thailand we studied height change over the last 3 decades.

**Methods:**

We analyzed height records for 33 000 21-year-old male military recruits, sampling 1000 per year from 1972 through 2006. We compared the height trend in Thailand to those noted in Europe, and discuss the former in the context of improvements in living circumstances in Thailand.

**Results:**

Over 35 years, mean height increased from 164.4 to 169.2 cm, an increment of nearly 5 cm. The height increase was negligible in the first decade (1972–1981), but substantially accelerated after that. In the period after 1990 the increase exceeded 3 cm. A similar overall height gain in Britain occurred over a much longer period (1750–1886).

**Conclusions:**

The increase in height among Thai men is biological evidence that a Thai health-risk transition—defined by both changing risks and outcomes—is well underway for height. Military recruits born during the 1960s through the 1980s had progressively healthier childhoods. Over this period child nutrition improved, infection and mortality rates declined, and preventive health services expanded. The combined effect of these factors is indicated by the increased adult height of Thai military recruits.

## INTRODUCTION

The population of Thailand has undergone a profound transformation in health risks and outcomes since World War II.^1^^–^^5^ High mortality and fertility rates have experienced declines^6^^,^^7^ that took much longer to occur in industrialized countries. Some of the reduction in mortality has been due to better medical care, ie, application of knowledge and technology that did not exist 100 years ago.^3^ However, the real impetus for this decrease in overall mortality in Thailand has been the substantial improvement in nutrition, and decreased rates of infection, in early childhood.

One way to evaluate progress in the environment of children is to investigate population trends in the height of young adults. Children grow to approximately 50% of their adult height by the age of 2 years; final adult height is strongly associated with the height a child attains in the first 2 to 3 years of life.^8^ Stunting during that period cannot be reversed by catch-up growth in later years, even if the child’s environment improves greatly.^9^ Therefore, at the population level, short adult height is indicative of poor nutrition and frequent infections in childhood, which are environmental factors that have grown less frequent in Thailand in the last 50 years.

Floud et al examined 2 centuries of height trends in countries that are now economically developed.^8^ This landmark study comprehensively analyzed historical height trends in relation to industrial development and is a fundamental benchmark for the measurement of population health. The most abundant data are derived from military recruit records, especially for Britain, where adult height began to rise during the industrial revolution (1750–1825). However, this early post-industrial trend did not continue and heights regressed during the next 50 years as the environment deteriorated in the 19th century slums. Around 1880, the height of young adult Britons began to increase again, although differentials by socioeconomic class persisted (ie, shorter stature among the poor). In the 210 years from 1770 to 1980, the mean height of male British military recruits increased 10 cm, from 165 cm to 175 cm. In other parts of Europe, there were similar increases. Height was lowest (159 cm) among military recruits from Austria and Norway in the mid-18th century, and highest (179 to 181 cm) among recruits from Norway and Holland in the 1980s. As both the lowest and highest heights were recorded in Norway, genetic influences may play less of a role than previously thought.

Floud et al concluded that adult height trends in the developed world reflected environmental and nutritional conditions during early childhood.^8^ To conduct similar research in Thailand—a country that began to industrialize only 50 years ago—we collected and analyzed height records of 33 000 21-year-old military conscripts who reported for duty in Bangkok during the period from 1972 through 2006. These recruits were born in the early period of Thai industrial growth (1951–1985). We discuss the observed trends in height in the context of the socioeconomic and environmental conditions that existed when the recruits were young children. This study was conducted in conjunction with other ongoing historical and prospective research on the health-risk transition in Thailand.^7^

## SUBJECTS AND METHODS

The modern Thai military recruitment law dates from 1917 and requires military service by all male citizens. A Reserve Officer Training Corps was established in 1935 and was adapted to the emerging education system in 1948. Students in the upper grades of high school, or in vocational schools or universities, were permitted to perform their military training on weekends. All other men were eligible for military conscription at age 21, and they are the population of the present study. The Thai Ministry of Defence allowed us to examine all archived records for Bangkok conscripts, which date back to 1972. Thus, the present study is based on the height records of 21-year-old men who were recruited for military service in Bangkok and its surrounding areas from 1972 through 2006. Data were missing for 1974 and 1997 and no information was available on the recruits’ education level or socioeconomic status.

Approximately 10 000 recruits per year were examined by the Ministry of Defence, and heights were recorded for those excluded from service for any reason, as well as for all accepted recruits. To determine the sample size needed to estimate average height for each observed year with 95% confidence limits of ±0.25 cm, we first gathered an initial sample of 705 heights and calculated their standard deviation (6.05 cm). We were then able to determine that the sample size needed was approximately 1000 per year, a sampling fraction of approximately 10%. Annual register books recorded heights for 7 examinees per page. Starting randomly, we selected every tenth page for 143 pages, thereby obtaining a sample of 1000.

Data were entered in an Excel spreadsheet and analyzed using SPSS software. To confirm that short examinees were not excluded, height distributions for the samples obtained in the first year and last year were checked against a normal curve. Mean heights plus standard deviation were calculated for each year, and mean annual increments were estimated by linear regression. Height trends were plotted as a smoothed moving average height curve and smoothed annual increments (or decrements) across the 35-year period. Finally, the historical 35-year height trend data were fitted to models using the curve estimation function of SPSS.

The Research and Development Institute of Sukhothai Thammathirat Open University (protocol 0522/10) and the Ethics Committee of the Australian National University (protocol 2004344) approved the study.

## RESULTS

**Figure 1.**
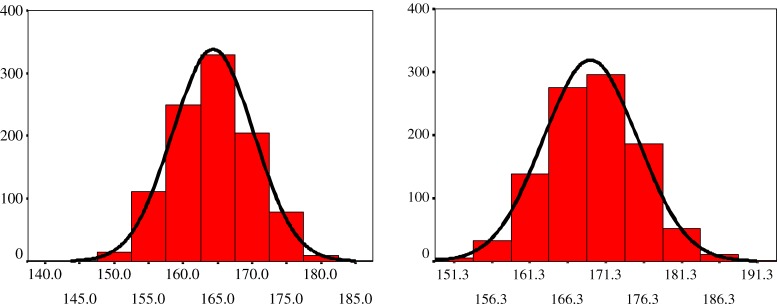
Distribution of height in cm among 21 year-old military recruits, 1972 and 2006. *n* = 1000 in each year; line shows normal curve for 1972 (left) and 2006 (right)

Figure 1 shows the height distributions of Thai military recruits at the start and end of the study period. Normal curves fit well to the distributions (skewness ≤0.02, kurtosis ≤0.02), which is statistical evidence that no height subgroup was excluded. The distributions also show that a population-wide height increase occurred over the 35 years observed and that the height distribution of 2006 was flatter and broader than that of 1972.

In 1972, the mean height of the 21-year-olds was 164.35 ± 5.89 cm. By 2006, mean height had increased to 169.23 ± 6.23 cm, a 4.88 cm increase (Table 1). Linear regression for height (ht) using the equation ht = a + bx, with x representing mean height in each investigated year (1972–2006), revealed a mean height increase of 0.134 cm per year.

**Table 1.** Mean height of 21-year-old male Thai military recruits, 1972–2006

Year	Mean height (cm)	Std. Deviation	Year	Mean height (cm)	Std. Deviation
	
1972	164.35	5.889	1990	166.02	6.199
1973	165.26	5.732	1991	165.30	5.959
1974	na*	na	1992	166.53	6.227
1975	164.79	5.842	1993	166.84	6.633
1976	165.35	5.779	1994	166.86	6.367
1977	164.93	5.842	1995	166.03	6.243
1978	164.94	5.747	1996	166.87	6.182
1979	164.75	6.023	1997	na	na
1980	165.86	5.954	1998	167.56	6.197
1981	164.92	5.726	1999	167.34	6.122
1982	165.23	6.021	2000	168.50	5.976
1983	164.85	5.800	2001	168.26	6.260
1984	165.29	6.407	2002	168.45	5.739
1985	165.65	5.721	2003	168.53	6.478
1986	165.94	5.591	2004	169.04	5.926
1987	165.65	5.680	2005	168.94	6.415
1988	166.23	6.015	2006	169.23	6.246
1989	166.05	6.007			

*data not available for 1974 and 1997.

**Figure 2.**
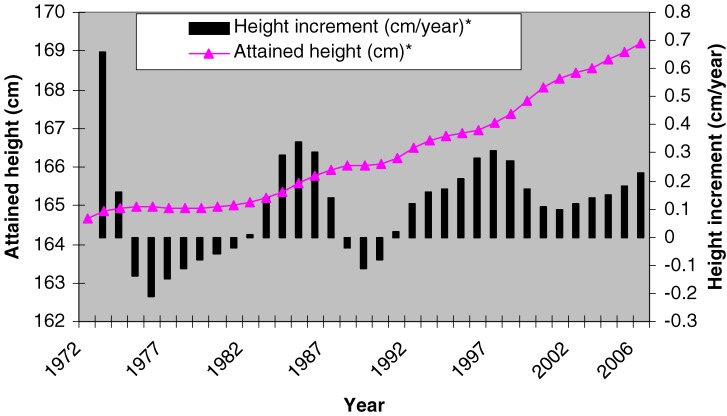
Mean attained height of 21 year-old Thai male military recruits, 1972–2006. *data smoothed by SPSS V11.5

To smooth the curve for mean attained height, a centered moving average was applied to the whole observation period, 1972 through 2006, and compared to the observed smoothed annual changes (Figure 2). The smoothed mean height of recruits increased little during the first decade, but the increase substantially accelerated in later years, most notably after 1990. The smoothed annual change in mean height was negative during 2 distinct periods: the 7-year period from 1954 to 1960 and the 3-year period from 1967 to 1969.

**Figure 3.**
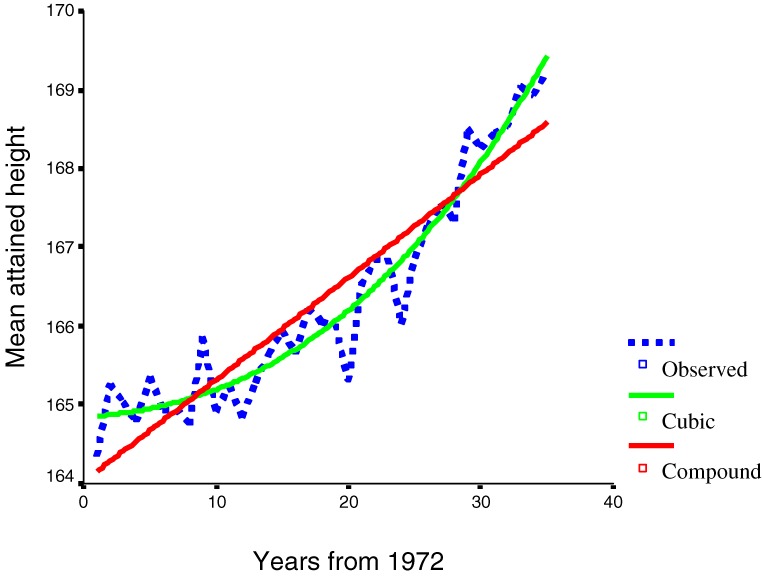
Fitted curves for Thai male military height increase over time from 1972

The 2 trend equations—the cubic and compound models—demonstrated a reasonably good fit for the historical 35-year height data (Figure 3). The equations and parameters are shown in Table 2. The compound model projection is well below the observed values near the end of the study period; the cubic model projection is slightly higher during the same period. It is possible that future gains in height will follow the trajectory of such historical curves.

**Table 2.** Equations for curves fitted to attained heights of male Thai military recruits, 1972–2006*

Parameters	Cubic model	Compound model
b0	164.8470	164.0280
b1	0.0096	1.0008
b2	0.0021	
b3	0.000 038	
t	number of years from 1972	number of years from 1972

Equations	Y = b0 + (b1 * t) + (b2 * t^2^) + (b3 * t^3^)	ln(Y) = ln(b0) + (ln(b1) * t)

*using curve estimation function of SPSS V11.5. Y is the attained height in cm.

## DISCUSSION

From 1972 through 2006 the average height of male 21-year-old military recruits in Bangkok increased from 164 cm to 169 cm, and the trend has accelerated since the mid-1980s. The rate of increase in attained height was more rapid in the last 20 years (3.5 cm increase) of the study period, and even more rapid in the last 10 years.

Our annual samples of the heights of military recruits were normally distributed, ie, short recruits were not excluded and random sampling succeeded in representing the whole group. Height measurements were made by trained military personnel and there is no reason to suspect that the measurements are inaccurate. Furthermore, 1979 data for military recruits show a similar mean height to that noted (165 cm) in the same year for a convenience sample of nearly 6000 young male residents of Bangkok.^10^ Thus, the trends noted in our study reflect those of the overall population of Bangkok.

The recruits studied are a less privileged group, because men of higher socioeconomic status were able to continue their education and thereby avoid conscription. However, secondary education in Thailand expanded rapidly in the 1990s, thus extending opportunities to perform weekend military service and avoid conscription. Therefore, it is possible that conscripts who were too poor to finish school were less prevalent in the last 10 years; however, there are no data to confirm this. It is also possible that the more affluent men had higher career aspirations and chose to avoid conscription more often than did less affluent men, during the final part of the observation period.

There was little increase in attained height during the first decade of observation (1972–1982), which indicates that there was limited improvement in child growth in the 1950s, when the recruits were small children. A child growth study conducted between 1952 and 1954 in a suburb of Bangkok supports this conclusion: after 6 months, nearly half of the children failed to gain weight, or even lost weight, due to malnutrition.^11^^,^^12^ Recruits born during and after the 1960s had a progressively better childhood health environment, as indicated by increases in adult height. Thai infant and maternal mortality rates in the early 1960s were approximately 85 per 1000 and 375 per 100 000, respectively; these rates decreased to approximately 25 per 1000 and 11 per 100 000, respectively, over the next 35 years.^2^

The observed height trend occurred during a period of extraordinary economic development in Thailand, during which GNP and real income increased continuously from 1958 to 1996. This economic prosperity lifted Thailand to middle-income status, greatly diminished extreme poverty, and ended the income stagnation that had lasted more than a century.^5^ Rising incomes enabled a corresponding rise in the general standard of living and culminated in an industrial export-led economic bubble that finally burst in 1997. The contraction was comparatively short-lived. Growth resumed after 2 years, but new challenges await as Thailand moves to a more skills-based economy.^13^

The height trend we observed reflects an overall increase in the standard of living, but it is also worth noting some specific improvements that changed the domestic environment during the period when the recruits were young children (the 1950s through the 1980s). During this 35-year period, many successful development strategies were implemented.^1^^,^^5^ Roads and transport were improved in the 1950s and 1960s, thereby expanding the food distribution network. Families became smaller once birth rates began to fall in the mid-1960s, particularly so after family planning was supported by the government, and by local and international nongovernmental organizations and aid agencies, from 1972; this made food more available within the family, and infection less common. When completion of primary school became the norm in the 1970s and completion of high school became common in the 1990s, parents were exposed to the modernizing influence of schools and teachers. Most households had acquired a fresh water supply, sanitation, electricity, radio, and television by the mid-1980s, and mobile telephones and comprehensive health services are now available to almost all Thais.

Advances in medical services and treatments played a direct role in the height increase. The mass health campaigns against malaria, yaws, smallpox, plague (1950s), and tuberculosis (1960s and 1970s) were highly successful in reducing the incidence of these diseases. Beginning in the mid-1970s, low-income parents have had access to primary health care, poor children have had a school lunch program, and poor pregnant and nursing women have been given food supplements and support from village health volunteers. Beginning in the late 1970s, most Thai infants have been immunized against tuberculosis, diphtheria, tetanus, and whooping cough.^14^^,^^15^

Adult height is the product of biological welfare, ie, it is the result of our socioeconomic and epidemiological environments during childhood.^16^ Height, labor productivity, and GDP are correlated in analyses of developed countries^17^; therefore, the increasing height of Thai military recruits may be both a cause and an effect of the rising Thai GDP. The Human Development Index (HDI) combines indices for life expectancy, education, and income and shows that by 2006 Thailand had reached an HDI rank of 74 in the world.^18^^,^^19^

After reaching a mean height of 164 cm, additional gains in the height of Thai conscripts have been much more rapid than those of their historical European counterparts. English men aged 21 years averaged 164.0 cm in height in 1750, but took considerably longer than 100 years to reach 168.5 cm, in 1886.^8^ Thai recruits experienced the same increase in height in just 35 years; if the present trend continues in Thailand, recruits will reach the current height of their counterparts in Europe (175–180 cm) in as little as 25 years. However, genetic differences may play a role in mean attained height. In addition, current and future childhood conditions in Thailand may prove less than optimal for obtaining maximum height.

Thai males are still far from reaching a mean height of 180 cm. Meanwhile, food availability has increased in Thailand. The combination of extra calories and short adult stature increases the risk of adult obesity, and it will be many more decades before short-statured Thai adults are no longer common. Therefore, a tendency towards frequent adult obesity, as noted 50 years ago among short adults of the British working class,^9^ will certainly affect Thais. Indeed, obesity has emerged as a new problem in Thailand over the last 15 to 20 years. This will exacerbate other known risk factors for child and adult obesity in Thailand, and lead to increased prevalences of associated conditions, such as diabetes.

The development strategies over the last 50 years in Thailand have increased the average height of young Thai citizens. However, the abrupt and severe economic downturn of 1997 quickly increased rates of child malnutrition.^4^ We must not forget that the height gains achieved in Britain when it first became industrialized regressed over a period of 50 years (1825–1875), due to worsening conditions brought about by urbanization. Thailand may soon experience a similar massive population shift, as only 40% of Thais currently live in cities. Therefore, we cannot be complacent about continuing height gains in the future.

Sustained efforts to ensure healthy childhoods in Thailand will be rewarded with stronger and taller citizens. Taller people live longer lives with less illness.^8^^,^^9^^,^^20^ On average, they will have gained more from their years of schooling as children, and will be more productive adults. The Thai government must preserve this fundamental goal for future development, so that all Thais can reach their full potential.
